# Dialyzer Reactions Revisited: A Fresh Perspective

**DOI:** 10.1016/j.xkme.2026.101475

**Published:** 2026-07-17

**Authors:** Charles Jun Han Ng, Roy Debajyoti Malakar

**Affiliations:** Department of Renal Medicine, Changi General Hospital, Singapore; Duke-NUS Medical School, Singapore

**Keywords:** Dialyzer reaction, biocompatibility, hypersensitivity, biomarker, allergy, tryptase, hemodialysis, complication, anaphylaxis

## Abstract

Dialyzer reactions can cause significant morbidity and even mortality. The current convention using time- and symptom-based classification has limited practical utility because of considerable overlap and diverse presentations. Several biomarkers have been described as diagnostic adjuncts, but clinical application remains sparse and inconsistent. There is also little guidance on subsequent management after diagnosis.

In this paper, we highlighted the diagnostic complexities and current limitations through 2 clinical cases. We hence proposed a novel, pragmatic clinical and biomarker-guided algorithm using serum tryptase levels to address these.

We adopted an acute increase from baseline levels of ≥ 20% + 2 ng/mL (20 + 2 rule) as the diagnostic threshold, demonstrated in one of the cases. Following this, ethylene oxide exposure should first be eradicated, as it remains one of the most common precipitants. If symptoms persist, decisions on dialyzer change should then be guided by clinical severity.

In conclusion, there is a dire need for better diagnostic precision and individualized management of dialyzer reactions. Our proposed algorithm can potentially fill this gap but requires validation in larger prospective studies. Future research could further refine the optimal diagnostic threshold and explore risk stratification using a similar clinical-biomarker approach for more precise management.

## Introduction

Dialyzer reactions are an important yet often overlooked complication of hemodialysis (HD), which can lead to significant morbidity and even mortality. These reactions can occur with any component of the extracorporeal circuit, but most cases in the literature are related to ethylene oxide (ETO),^1^ a sterilant used for dialyzers and tubing, and non-biocompatible cuprophan membranes.^2^ Other known triggers include the use of angiotensin-converting enzyme inhibitors with acrylonitrile dialyzers, and formaldehyde used for dialyzer reprocessing.^3^ Despite the global shift over the years toward more biocompatible synthetic membranes and the use of alternative sterilization methods, such as steam or irradiation, there is still a substantial burden, and dialyzer reactions remain a major clinical concern.^4^^,^^5^

**Figure 1:**
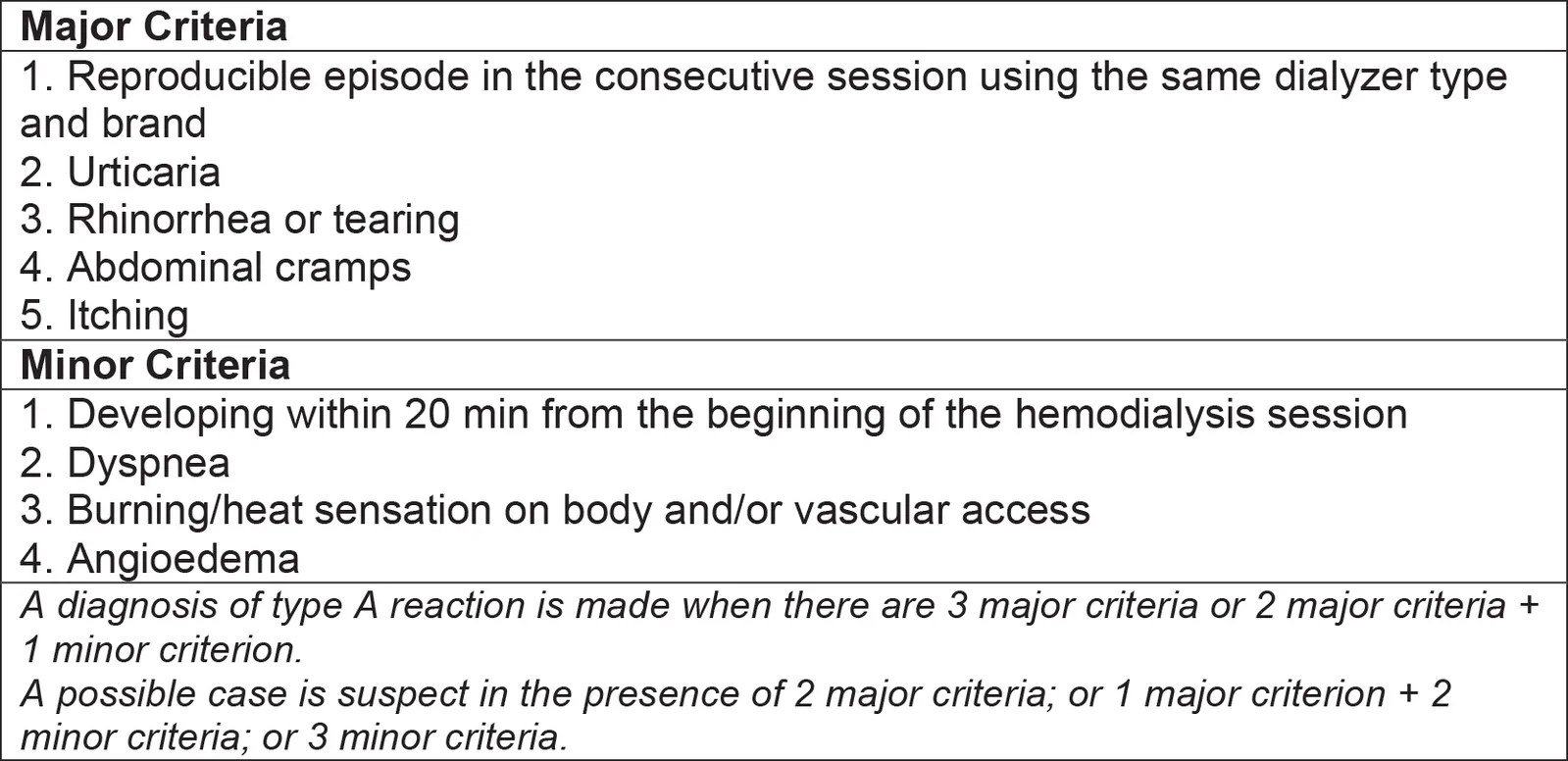
Daugirdas criteria for diagnosis of type A reactions.

Historically, Daugirdas and Ing classified dialyzer reactions into types A and B based on onset and severity of symptoms with proposed pathophysiological mechanisms. Type A reactions typically begin within 20 minutes of dialysis initiation with variable symptoms, ranging from itch to cardiorespiratory arrest. These may be anaphylactic (immunoglobulin E [IgE]-mediated), typically caused by ETO and formaldehyde,^3^ or anaphylactoid (non-IgE mediated) and can be diagnosed based on the Daugirdas criteria (Fig 1).

Type B reactions, on the other hand, are more common and tend to be milder. They occur later during the dialysis session and are attributed to complement activation, usually because of less biocompatible membranes. Symptoms are nonspecific, such as back pain, but can also manifest as dyspnea and hypotension.^6^

Evidently, there is considerable overlap between the two in terms of symptoms and onset timing, hence limiting practical utility. Adding to the clinical dilemma, presentation is diverse, not limited to the above-described symptoms, and these can easily masquerade as any other acute conditions in hospitalized patients, such as infection or cardiac event, resulting in delayed or missed diagnoses.

Several biomarkers have been described as potential diagnostic adjuncts to address this, but clinical application remains sparse and inconsistent.^7^ There is also little guidance on subsequent management after diagnosis. Although most case reports demonstrated resolution after changing the dialyzer to cellulose triacetate,^4^^,^^5^ a modified cellulose membrane, it may not be prudent to do so for all patients with dialyzer reactions.

As such, there is clearly a gap and a dire need for better diagnostic precision and individualized management. In this paper, we highlighted the limitations of current classification through 2 clinical cases and hence proposed a novel, pragmatic clinical and biomarker-guided algorithm using serum mast cell tryptase levels to address these.

## Exemplifying the Diagnostic Dilemma and Current Limitations

### Case 1: Eosinophilia

Case 1 is a 71-year-old female with chronic kidney disease stage 5 secondary to hypertensive nephrosclerosis, admitted electively for total parathyroidectomy with deltoid reimplantation for primary hyperparathyroidism. Other significant past medical history includes osteoporosis and previous cerebrovascular accident. Postoperatively, her kidney function deteriorated further; hence, she was initiated on long-term HD via a tunneled dialysis catheter using a Gambro AK 98 system with Polyflux 170H dialyzer (Baxter).

She tolerated the first 3 sessions, but developed intradialytic hypotension subsequently, requiring fluid boluses and dialysis termination. This became progressively more severe and occurred earlier during dialysis with each session, initially at 2 hours upon initiation to as early as 15 minutes eventually. There were no other symptoms or signs of anaphylaxis.

Concurrently, she developed recurrent low-grade fevers mainly occurring post-dialysis with a mild exanthem starting from her third session despite receiving broad-spectrum antibiotics. Extensive work-up, including microbiological cultures, imaging, and autoimmune screen, all returned negative. Tunneled dialysis catheter exchange was also done, but there was no defervescence. Her intradialytic hypotension was investigated with a transthoracic echocardiogram, which showed normal ejection fraction, and telemetry did not reveal any significant arrhythmia. A short synacthen test was also performed, which demonstrated an adequate response.

**Figure 2:**
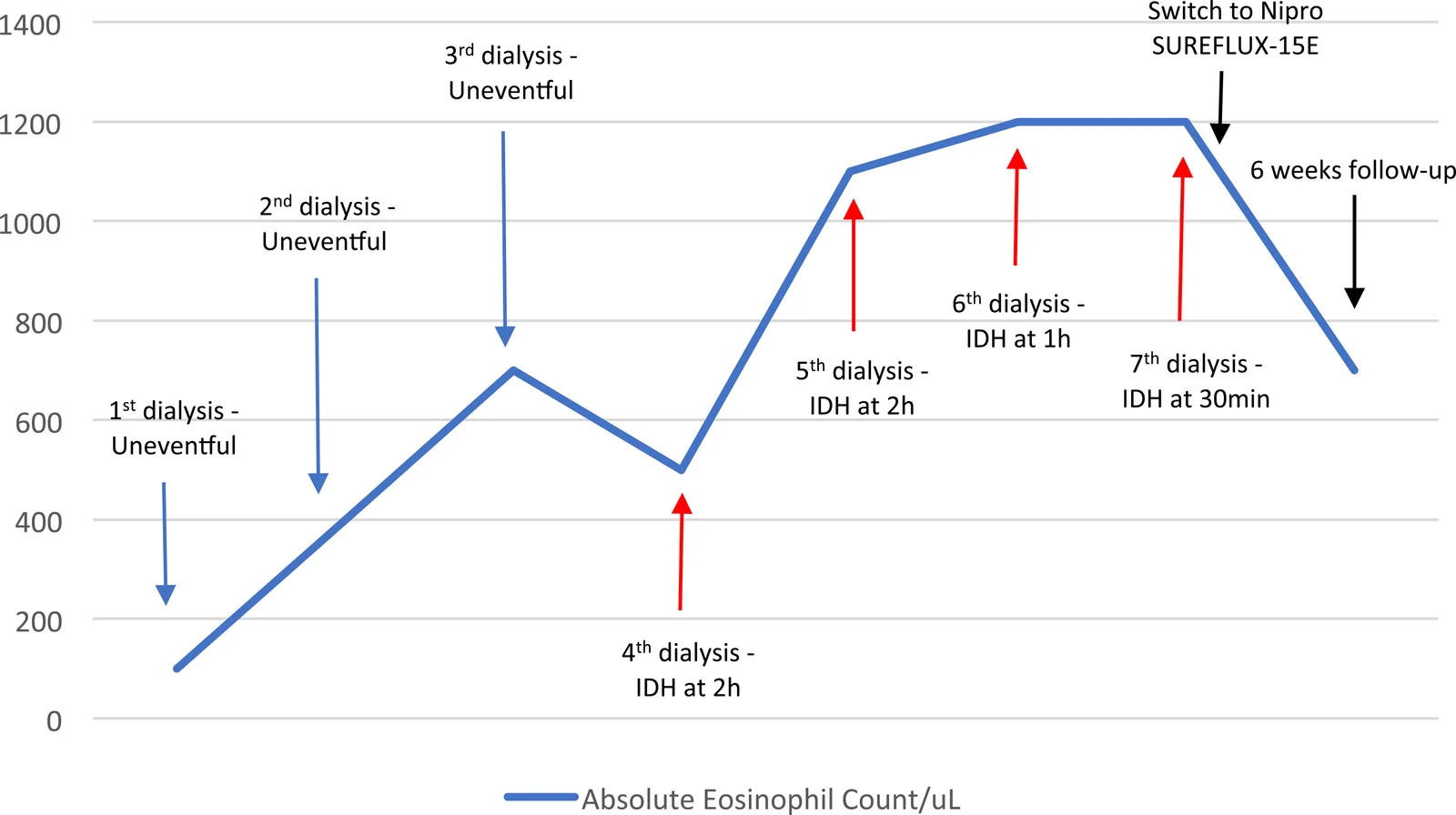
Absolute eosinophil count in relation to events (case 1).

Given the overall clinical scenario, coupled with rising peripheral eosinophilia, a dialyzer reaction to Polyflux was suspected, and she was switched to a SUREFLUX-15E dialyzer (Nipro), a cellulose triacetate membrane. She tolerated HD thereafter with no further episodes of intradialytic hypotension, and her fever subsided. At the 6-week follow-up, there was a notable improvement in her eosinophil count (Fig 2).

### Case 2: Mast Cell Degranulation Without Eosinophilia

Case 2 is a 75-year-old male with kidney failure secondary to diabetes mellitus on chronic HD via tunneled dialysis catheter. He was admitted for shortness of breath with desaturation occurring 1 hour into dialysis, requiring termination and salbutamol rescue. Other significant past medical history includes hypertension, well-controlled asthma on maintenance inhalers, and ischemic heart disease. Serial electrocardiogram and troponins were not suggestive of acute coronary syndrome, and the chest radiograph was unremarkable.

His symptoms abated with bronchodilators and systemic corticosteroids; however, these recurred in-hospital thirty minutes into dialysis using an AK 98 system with a Polyflux 170H dialyzer. Retrospective review of clinical records showed no major issues with the same setup when he was first initiated on chronic HD, except for the eighth session where he developed mild shortness of breath toward the end, but this was attributed to an asthma exacerbation and was relieved with salbutamol. Subsequently, upon discharge to the community dialysis unit, corroborative history revealed that he had been having recurrent shortness of breath and wheezing occurring 30 minutes to 1 hour into dialysis, which would usually abort with rescue salbutamol. His latest episode was, however, refractory and associated with desaturation, hence warranting admission. Hemodynamics remained stable otherwise, and there was no rash or angioedema. The Fresenius 5008 system with a FX 80 CorDiax dialyzer (Fresenius Medical Care) was used at the community dialysis unit.

In view of the above, an anaphylactoid-type dialyzer reaction was suspected, and serum mast cell tryptase was sent, which returned significantly elevated at 44.1 ng/mL (normal adult level < 11.4 ng/mL). A repeat test was done the next day, which showed improvement to 15.6 ng/mL, suggesting mast cell activation during the event. He was then switched to a SUREFLUX-15E dialyzer and tolerated HD afterward with no further recurrence of symptoms. Interestingly, his absolute eosinophil count remained normal throughout these episodes.

As shown in Table 1, both cases had no ethylene oxide exposure because all dialyzers and tubing were sterilized by either steam or irradiation. They were also admitted at different time points and dialyzed with different machines using ultrapure fluids; hence, reactions were unlikely because of pyrogenic substances from contaminated dialysate. In addition, neither of them had any symptom recurrence after switching to a cellulose triacetate membrane, supporting the diagnosis of dialyzer reaction to synthetic membranes.

**Table 1:** Summary of Illustrated Cases With Various Dialysis Setups and Respective Reactions

Dialyzer	Membrane	Potting/housing	Membrane sterilization	Machine	Tubing	Reaction
Case 1: Eosinophilia						
Polyflux 170H	Polyarylether-sulfone/polyvinyl-pyrrolidone/polyamide	Polyurethane/polycarbonate	Steam	Gambro AK 98	NovaLine (Baxter), gamma irradiated	Hypotension, Fever, Exanthem
SUREFLUX-15E	Cellulose triacetate	Polyurethane/polypropylene	Gamma irradiation	Gambro AK 98	NovaLine, gamma irradiated	-
Case 2: mast cell degranulation without eosinophilia						
Polyflux 170H	Polyarylether-sulfone /polyvinyl-pyrrolidone /polyamide	Polyurethane/polycarbonate	Steam	Gambro AK 98	NovaLine, gamma irradiated	Shortness of breath with wheezing, Desaturation
FX 80 CorDiax	Helixone plus (Polysulfone)	Polyurethane/polypropylene	Steam	Fresenius 5008	LifeLine Beta 5008 (Fresenius), e-beam irradiated	Shortness of breath with wheezing, Desaturation
SUREFLUX-15E	Cellulose Triacetate	Polyurethane/polypropylene	Gamma irradiation	Gambro AK 98	NovaLine, gamma irradiated	-

These cases illustrated that dialyzer reactions can present variably, making it notoriously difficult to tease out, especially in patients admitted for other acute issues because there can be overlap in symptoms and many differentials for each presentation. There was display of priming and sensitization with repeated exposures, evidenced by increasing severity and/or earlier manifestation of symptoms during dialysis. Case 2 also demonstrated cross-reactivity among members of the polyarylsulfonate family, namely polyarylethersulfone and polysulfone. This is consistent with literature reporting significant cross-reactivity in about 85% of the cases who were switched to different polysulfone or polyethersulfone dialyzers.^7^

More imperatively, they reiterated significant limitations of the traditional time and symptom-based model. Using current convention, only case 1 would have fulfilled the definite diagnosis of type A reaction, and this could only be made much later in the course after repeated sensitization, resulting in significant morbidity. Case 2, on the other hand, would have been missed if not for a very high index of clinical suspicion. This definitely leaves much to be desired in our current era of modern precision medicine. Furthermore, there is no guidance on subsequent management even after diagnosis is clinched.

## A Fresh Perspective

**Figure 3:**
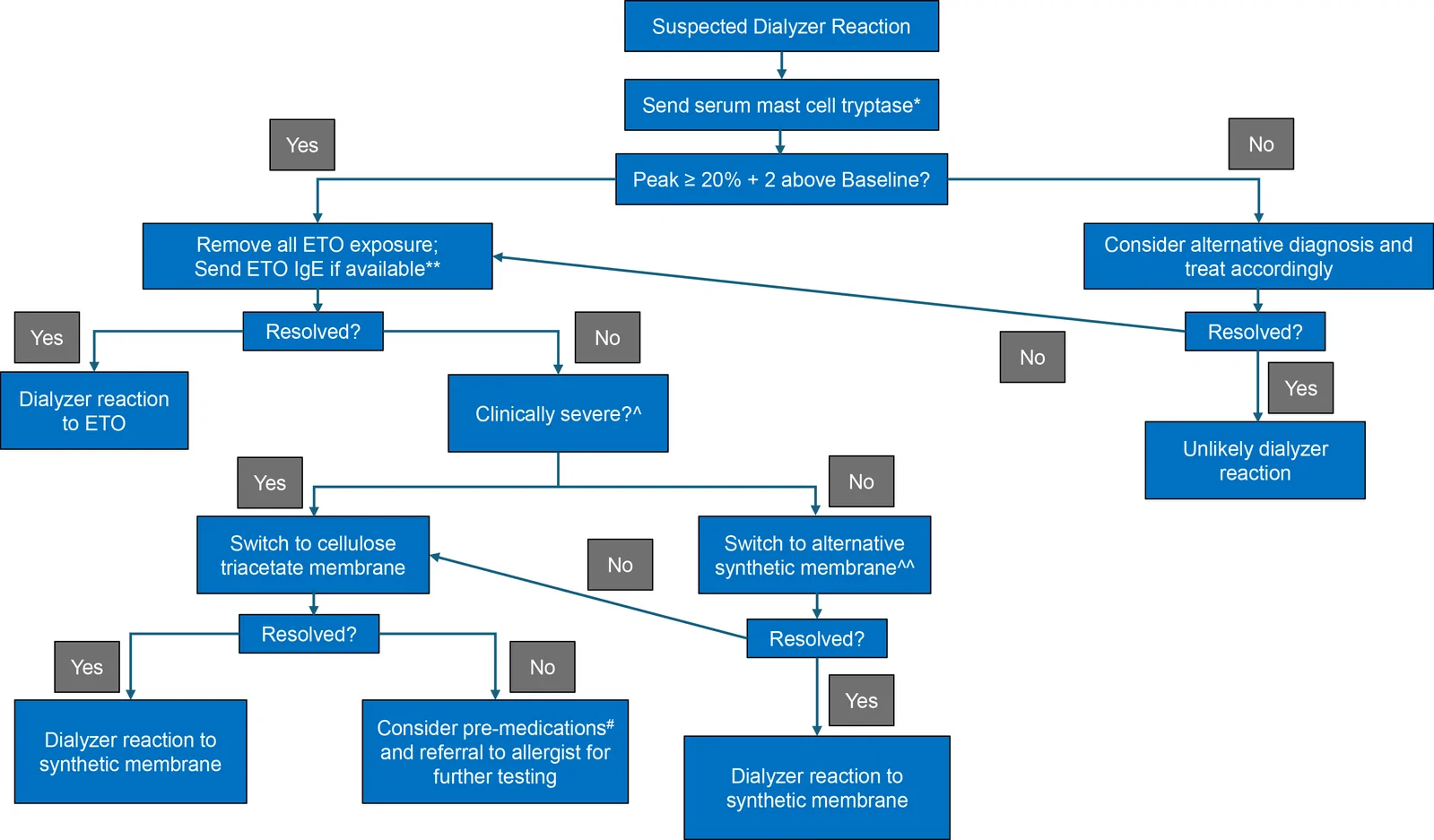
Proposed novel algorithm for suspected dialyzer reaction. ∗Send within 1 to 4 hours of symptom onset (peak) and at least 24 hours after complete resolution (baseline). ∗∗Review sterilant in entire extracorporeal circuit, including fistula needles, tubing, and dialyzers. ˄Clinically severe defined as hemodynamic and/or respiratory compromise, ie, systolic blood pressure < 90 mmHg, mean arterial pressure < 65 mmHg and/or hypoxemia. ˄˄Avoid switching within the same family because of significant cross-reactivity. ^#^Premedications include antihistamines and/or corticosteroids.

We proposed a novel, pragmatic clinical and biomarker-guided algorithm using serum levels of mast cell tryptase for better diagnostic precision and individualized management (Fig 3).

When selecting a suitable biomarker as a diagnostic adjunct for dialyzer reactions, several key factors must be considered. The biomarker should show a strong correlation or trend during the reaction, have a short half-life, and remain largely unaffected by dialysis treatment. These characteristics will aid accurate and early diagnosis.

### Serum Mast Cell Tryptase

Tryptases are serine proteases found in all mast cell phenotypes, with small amounts detected in basophils (∼0.4% of that in mast cells). Basal serum tryptase levels reflect total mast cell burden, with an acute rise occurring during mast cell activation and degranulation in response to allergens or downstream effects of complement activation.^8^ Advanced chronic kidney disease is associated with higher basal levels because of increased serum stem cell factor.^9^ Fortunately, serum tryptase has a reasonably short half-life of 2 hours, with peak levels occurring around 30 minutes to 2 hours after onset of symptoms.^10^ It also has a large molecular weight of 134 kDa, rendering it nondialyzable. These important properties allow us to determine baseline levels during the typical interdialytic period of 48-72 hours to discern retrospectively whether there was a significant rise during the reaction. Expert consensus recommends testing within one to four hours of a suspected mast cell activation event (peak) and at least 24 hours after resolution of all symptoms (baseline).^11^ An acute increase from baseline levels of ≥ 20% + 2 ng/mL (20 + 2 rule) strongly supports the diagnosis,^12^ which has 75% sensitivity and 86% specificity in diagnosing anaphylaxis,^13^ neatly demonstrated in case 2 (peak level, 44.1 ng/mL; baseline level, 15.6 ng/mL). We had concerns about whether this could be confounded by other atopic conditions, such as asthma, which might coincidentally flare up during dialysis. However, most available data show poor association between serum tryptase levels and asthma exacerbation because mast cell degranulation occurs locally in the lungs, resulting in high tryptase levels in bronchoalveolar lavage but not to the same extent in the circulation.^14^

We considered other potential biomarkers described in the literature, but they have major limitations:

#### Histamine

Histamine is released by mast cells during hypersensitivity reactions and correlates with severity. However, there is no well-defined threshold for the diagnosis of anaphylactic or anaphylactoid reactions. It also has a very short half-life of 20 minutes, requiring sample collection within the first hour of symptom onset, which may be challenging. In addition, histamine testing is not readily available in all centres and requires special handling during sample transport to avoid breakdown, limiting its widespread use.^15^

#### Eosinophil Count

Eosinophil count is another purported biomarker and is commonly raised in atopic disorders, as well as hypersensitivity reactions. However, it can also be elevated in certain infections (typically parasitic) and many other conditions, hence lacking specificity. It has a rather long circulating half-life of 6-12 hours, which is not ideal for detecting trends during the interdialytic period. The decrease in eosinophil count post-exposure removal does help support the diagnosis retrospectively, as seen in case 1 after switching dialyzers, but is not useful for clinical decisions. Furthermore, they are mainly stimulated by IgE, which might potentially miss anaphylactoid (non-IgE-mediated) and complement-mediated reactions.^16^ This findings likely explains why there was no correlation with eosinophil count for case 2.

#### Total Serum IgE

Elevated total serum IgE levels during dialyzer reactions have been widely demonstrated, and the presence of allergen-specific IgE can help confirm the culprit.^1^^,^^4^ However, the latter is not available for all allergens, except for ETO in some centres, and false-negative results have been reported up to 3 and a half weeks post exposure,^17^ making it only useful as a rule-in test. Similar to eosinophil count, the half-life of serum IgE is long at 2 to 3 days, hence not suitable for interdialytic trending and only supports retrospective diagnosis post-exposure removal. It also lacks sensitivity with limited yield for non-IgE-mediated reactions.^18^^,^^19^ There is a potential role for anti-IgE therapy, such as omalizumab, in patients with persistently elevated levels and recurrent dialyzer reactions with no identifiable precipitant, but this is more relevant for refractory cases.^20^

Following diagnosis, we recognize that ethylene oxide is still one of the most common triggers^1^ for dialyzer reactions, and it remains widely used today as a sterilant in dialyzers, fistula needles, and dialysis tubing. As such, ETO should first be eradicated by thorough rinsing of the extracorporeal circuit before patient connection and switching to an alternative sterilant, such as steam or irradiation, especially if positive for ETO IgE.^21^ As mentioned previously, the absence of ETO IgE, particularly within the first 2 weeks, does not exclude ETO as a potential trigger, given the high false negative rate during this early period.^17^

If symptoms persist, the subsequent decisions on dialyzer changes should be based on clinical severity. For severe reactions causing hemodynamic and/or respiratory compromise, ie, systolic blood pressure < 90 mmHg, mean arterial pressure < 65 mmHg, and/or hypoxemia, we suggest an immediate switch to a cellulose triacetate membrane to avoid further morbidity. For mild to moderate reactions, clinicians can first change to an alternative synthetic membrane. This should be of a different family because of significant cross-reactivity, especially within the polysulfone group.^7^ This approach has been shown to be effective in some cases^22^ and reduces indiscriminate change to non-synthetic membranes. If unresolved, then switching to a cellulose triacetate membrane is reasonable. In refractory cases, pre-medications, such as antihistamines, can be considered, preferably avoiding corticosteroids because of bone and metabolic complications. An allergist referral is also warranted for further testing.

In this paper, we highlighted the diagnostic complexities and limitations of current conventions through 2 case illustrations, stressing the need for a change in clinical practice. To address these, we introduced a novel, pragmatic clinical and biomarker-guided algorithm using serum levels of mast cell tryptase. This algorithm is easy to implement and provides clear guidance for timely diagnosis and management of dialyzer reactions.

There are a few limitations to this algorithm. First, testing for serum tryptase levels may not be readily available in all centers. Second, the adopted diagnostic threshold can miss dialyzer reactions that do not cause massive mast cell degranulation, such as those mediated via contact system activation. This limits overall sensitivity. However, serum tryptase levels strongly correlate with clinical severity^23^; hence, cases that do not meet criteria are less likely to cause significant morbidity. Furthermore, although this algorithm is primarily designed as a rule-in approach to minimize overdiagnosis and unnecessary membrane change, it still allows inclusion of these “missed” cases if symptoms persist despite investigation and treatment of alternative diagnoses. Lastly, the “20 + 2 rule” has not been extensively studied or validated specifically for dialyzer reactions. In the largest prospective study by Rodríguez et al^24^ examining acute and baseline tryptase levels in hypersensitivity reactions to polysulfone-based membranes, none met the proposed diagnostic criteria. However, sample size was small (n=20) and limited to a Spanish population; thus, genetic variations could have confounded the results, possibly because of differences in α- and β-tryptase-encoding gene copies.^25^ Although the “20 + 2 rule” remains the most widely accepted threshold for diagnosing mast cell activation syndrome and anaphylaxis, it undoubtedly requires further validation for dialyzer reactions in larger and ethnically diverse cohorts.

In conclusion, dialyzer reactions can present variably and atypically; hence, clinicians should always maintain a high index of suspicion in patients receiving HD, particularly those with persistent intradialytic symptoms despite appropriate treatment of other acute conditions. There is a dire need for better diagnostic precision and individualized management of dialyzer reactions. Our proposed algorithm can potentially fill this gap but requires validation in larger prospective studies. Future research could further refine the optimal diagnostic threshold, given higher baseline tryptase levels in advanced chronic kidney disease, and even explore risk stratification using a similar clinical-biomarker approach for more precise management.
